# Is There an Optimal Timing of Adductor Canal Block for Total Knee Arthroplasty?—A Retrospective Cohort Study

**DOI:** 10.3390/jpm11070622

**Published:** 2021-06-30

**Authors:** Yan-Yuen Poon, Johnson Chia-Shen Yang, Wen-Yi Chou, Hsiao-Feng Lu, Chao-Ting Hung, Jo-Chi Chin, Shao-Chun Wu

**Affiliations:** 1 Department of Anesthesiology, Kaohsiung Chang Gung Memorial Hospital and Chang Gung University College of Medicine, Kaohsiung 833401, Taiwan; elephant423@gmail.com (Y.-Y.P.); maple@cgmh.org.tw (H.-F.L.); timtim0070@gmail.com (C.-T.H.); 2 Division of Plastic and Reconstructive Surgery, Department of Surgery, Kaohsiung Chang Gung Memorial Hospital and Chang Gung University College of Medicine, Kaohsiung 833401, Taiwan; johnson.c.yang@gmail.com; 3 Department of Orthopedic Surgery, Kaohsiung Chang Gung Memorial Hospital and Chang Gung University College of Medicine, Kaohsiung 833401, Taiwan; murraychou@yahoo.com.tw; 4 Department of Anesthesiology, Park One International Hospital, Kaohsiung 813322, Taiwan; jochi731@gmail.com

**Keywords:** adductor canal block, total knee arthroplasty, visual analogue scale, continuous passive motion, postoperative pain

## Abstract

Adductor canal block (ACB) has gained popularity for postoperative pain control after total knee arthroplasty (TKA). However, its role in TKA has been questioned recently. Our study aimed to clarify the role of ACB in reducing postoperative pain after TKA and to elucidate an optimal timing to perform ACB for better outcomes. We conducted a comprehensive review of the perioperative records of 652 patients undergoing primary TKA from January 2019 to December 2019. Patients were divided into three groups: Group A received general anesthesia without ACB, Group B received ACB before inducing general anesthesia, and Group C received ACB at the post-anesthesia recovery unit (PACU). Patients in Groups B and C had lower pain visual analogue scale (VAS) scores than patients in Group A at the PACU. Opioid consumption was similar among the three groups; however, a slightly higher dose was required by Group A patients. Higher VAS scores were recorded in the ward in Group A than in Groups B and C with the leg at rest. In addition, higher VAS scores were recorded in Group A than in Groups B and C with the leg in continuous passive motion (CPM) training. More patients in Group A (34.9%) quit their first CPM training after a few cycles than those in Groups B (27.0%) and C (20.1%). Group A patients required a higher per kg dose of opioids in the ward than Groups B and C patients. Additionally, the hourly consumption of sevoflurane was similar among the three groups of patients, while Group A and C patients required a higher hourly per kg dose of intraoperative opioids than Group B patients. More patients in Group A (67.6%) and C (61.7%) developed intraoperative hypertension than patients in Group B (52.7%). There was no significant difference in PON (postoperative nausea), POV (postoperative vomiting), postoperative dizziness, or patient satisfaction among the three groups of patients. Group A patients had a longer length of hospital stay compared to Group B and C patients. In conclusion, preoperative ACB could be a better choice for patients undergoing TKA as it decreases intraoperative opioid consumption and facilitates a stable hemodynamic state during surgery.

## 1. Introduction

Population aging is a human success story; however, it is estimated that the number of people aged 65 years and above would be 1.5 billion in 2050—that is, one in six people in the world will be aged 65 years and above in 2050 [1]. Aging is associated with many chronic diseases, including degenerative diseases and cancer [2]. Osteoarthritis (OA) is strongly associated with aging, and OA is one of the leading causes of physical disability in the elderly [3]. Taiwan has been an “aged society” since 2018, as over 14% of the population is 65 years of age or older [4]. A recent retrospective study showed that 154,553 total knee arthroplasties (TKAs) were performed in Taiwan between 1996 and 2010, and the number of TKAs increased from 26.4 to 74.6 per 100,000 citizens during this period [5]. An immense global demand for TKA is anticipated due to prolonged life expectancy in most developed and developing countries. An 85% increase is expected in the number of primary TKAs in the United States by 2030 [6].

The first ivory TKA performed on a 17-year-old woman in 1890 by a German surgeon, Dr. Gluck, opened a new era of surgical treatment for a completely dysfunctional knee joint in orthopedic surgery [7]. It was not until the early 1950s that the prosthetic materials and anatomic designs had greatly improved. Recently, a close collaboration between surgeons and engineers has further excelled the role of artificial knee joints by improving patients’ quality of life through pain relief and restoration of knee joint function. TKA is generally regarded as an effective treatment for severely degenerative OA knees with excellent surgical outcomes [8]. Despite great advances in either surgical technique or prosthesis design [9], acute postoperative pain in TKA patients remains an important issue for surgeons. It has been estimated that over 60% of patients experience severe pain after TKA [10,11]. Adductor canal block (ACB), a relatively novel technique, was first introduced by Lund et al. [12] in 2011 in an attempt to relieve postoperative pain after major knee surgery. In recent years, ultrasound-guided ACB has gained popularity as a technique for postoperative pain control in TKA patients [13]. However, a recent systematic review [14] concluded that it was uncertain whether patients treated with ACB had a lower pain intensity, fewer opioid-related adverse events, and fewer accidental falls during postoperative care compared to those given sham treatment or compared to those treated with a femoral nerve block. The uncertainty of ACB’s role in reducing postoperative pain in TKA patients formed the impetus for our study with the primary aim of assessing three important issues. First, to determine if ACB could effectively reduce postoperative pain after TKA in elderly patients. Second, to clarify if the timing of ACB administration—preoperatively or postoperatively—affects the patient’s outcome. Third, to determine the extent to which preoperative ACB affects the usual practice of general anesthesia. This study aimed to answer these three questions through a comprehensive review of pre-anesthesia, anesthesia, post-anesthesia recovery care unit (PACU) and of postoperative visit records of patients who underwent primary TKA under general anesthesia.

## 2. Materials and Methods

This study was approved by the Institutional Review Board of Kaohsiung Chang Gung Memorial Hospital (IRB number: 202100276B0). Informed consent was waived due to the retrospective nature of the study. All methods were performed in accordance with the relevant guidelines and regulations. The medical and anesthesia records of patients who underwent primary TKA between January 2019 and December 2019 were retrieved from the hospital’s electronic database. Data during the PACU stay, and data from routine postoperative daily visits, which were performed by well-trained nurse anesthetists within 24 h after surgery, were also reviewed. The exclusion criteria included spinal anesthesia, desflurane anesthesia, anesthesia without bispectral index monitoring (BIS), and records with missing data.

The patients were divided into three groups: patients in Group A received general anesthesia only, patients in Group B received preoperative ACB before inducing general anesthesia, and patients in Group C received postoperative ACB at the PACU. General anesthesia was induced using Propofol (1–2 mg/kg) as a standard practice in our hospital [15]. The use of rocuronium (1 mg/kg), cis-atracurium (0.2 mg/kg), alfentanil (10 mcg/kg), and sevoflurane (1–1.3 MAC) depended on the anesthesiologist’s preferences, and a fresh gas flow of 50% oxygen with air was maintained at 1 L/min. The BIS score was maintained in the range of 40–60 during anesthesia. ACB was performed as described in our recent study [15], using an ultrasound-guided technique with a total injection volume of 21 mL, which was a mixture of 10 mL 0.5% levobupivacaine, 5 mL 2% lidocaine, 5 mL normal saline, and 1 mL dexamethasone. The timing of preoperative or postoperative ACB depended on the anesthesiologist’s preference. Options for postoperative pain control included intravenous opioids and intravenous parecoxib. The visual analog scale (VAS, 0–10) was used to assess the postoperative pain response. The VAS score was also used as an indicator of the efficacy of the pain treatment modality in the ward. VAS scores were obtained when patients were at rest or during continuous passive motion (CPM) training. The length of stay after surgery and patient satisfaction (1–5) were also recorded upon discharge from the PACU and hospital. 

### Statistical Analysis

Numerical variables were tested using one-way analysis of variance and expressed as medians (interquartile range (IQR)). Bonferroni correction was used for the post-hoc analysis. The chi-square or Fisher’s exact test was used to analyze the categorical variables. Statistical analyses were performed using SPSS (version 22.0; IBM Corp., Armonk, NY, USA). Statistical significance was set at *p* < 0.05.

## 3. Results

**Figure 1:**
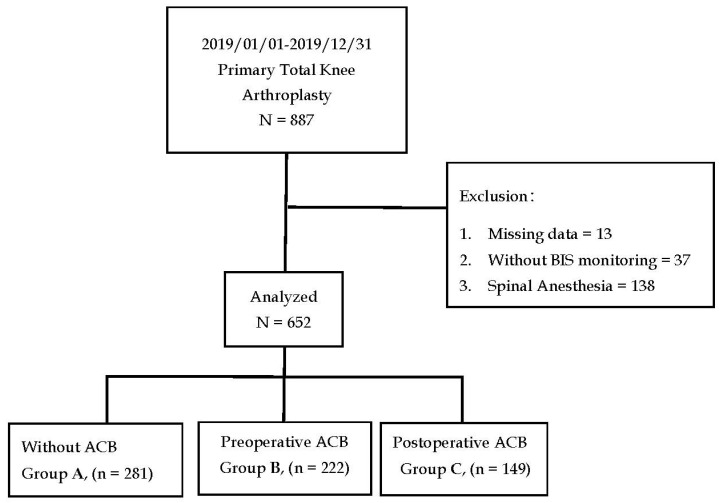
Flow chart of the allocation of patients who underwent primary total knee arthroplasty into Group A (non-ACB), Group B (preoperative ACB), and Group C (postoperative ACB).

A total of 887 medical and anesthesia records of patients who underwent primary TKA were retrieved from the hospital’s electronic database. After exclusion, we included 652 patients for statistical analyses. We deliberately excluded patients who underwent desflurane anesthesia because the number of cases was comparatively lesser (n = 47). Patients were segregated into Group A (n = 281), Group B (n = 222), and Group C (n = 149), as shown in Figure 1. Table 1 summarizes the demographic characteristics of the patients. There was no significant difference in the distribution of sex, ASA physical status, or comorbidities among the three groups. Age, body weight, and anesthesia time were similar among the three groups of patients. 

The effects of ACB on the intraoperative and postoperative courses are summarized in Table 2. Hourly consumption of sevoflurane (mL/kg/h) was similar among the three groups; the median consumption levels in Group A, Group B, and Group C were 0.21 (0.17–0.26) mL/h, 0.20 (0.17–0.25) ml/h, and 0.21 (0.17–0.26), respectively. We converted all of the opioids used in the study into a unified unit [16] and milligram morphine equivalent (MME) for comparison among the three groups. The intraoperative hourly per kg opioid consumption was significantly higher in Groups A and C than in Group B—0.078 (0.059–0.098) MME, 0.078 (0.062–0.092) MME, and 0.065 (0.048–0.088) MME, respectively. The incidence of intraoperative hypertension (systolic blood pressure > 30% of baseline) was significantly higher in Groups A and C than in Group B (67.6% and 61.7% versus 52.7%, respectively). In the PACU, a slightly higher opioid consumption was noted in Group A than in Groups B and C; however, the difference was not significant. There was a significant difference in the VAS scores among the three groups upon discharge from the PACU (4.0 (4.0–4.0), 2.0 (2.0–2.0), and 1.0 (1.0–1.0), respectively (Table 2)). Higher patient satisfaction was recorded in Group C patients upon discharge from the PACU. A significantly higher per kg opioid consumption was found in Group A than in Groups B and C in the ward, 0.134 (0.113–0.156) MME, 0.120 (0.101–0.139) MME, and 0.104 (0.087–0.121) MME, respectively. VAS scores were assessed at two different instances in the ward: with the leg in the resting state and the leg in continuous passive motion (CPM). The VAS scores were significantly higher in Group A than in Groups B and C in the leg resting state, 3.0 (3.0–5.0), 2.0 (1.0–2.0), and 1.0 (1.0–2.0), respectively. In addition, significantly higher VAS scores were recorded in Group A than in Groups B and C while undergoing CPM training—3.0 (3.0–5.0), 2.0 (2.0–3.0), and 2.0 (1.0–2.0), respectively. CPM was routinely performed 8 h after TKA; more patients in Group A than in Groups B and C refused to continue CPM training due to intolerable CPM-associated pain (34%, 27.0%, and 20.1%, respectively). There was no significant difference in PON, POV, postoperative dizziness, or patient satisfaction among the three groups. No accidental falls or ACB-related complications were observed. Patients in Group A had a significantly longer length of stay as compared with those of Groups B and C—4.5 (4.0–5.0) days, 4.0 (3.5–5.0) days, and 4.0 (3.5–5.0) days, respectively.

## 4. Discussion

TKA is generally considered an effective treatment for end-stage knee OA [17]. However, patients can suffer from severe postoperative pain [10,11] after surgery due to extensive bone resection [18] and soft tissue manipulation [19,20]. ACB has gained popularity for the treatment of postoperative pain in TKA patients in the recent years [14,21,22]. One of the interesting findings revealed in this study was that the hourly consumption of sevoflurane was similar among the three groups of patients, despite preoperative ACB being performed in Group B patients. This raised the fundamental question of whether preoperative ACB could alleviate surgical pain during surgery. This result was in contrast to our recently published study [15] that patients who received preoperative ACB consumed less sevoflurane than those who did not receive ACB in knee arthroscopic surgery. One might consider that TKA involves a broader area of bone and tissue destruction, and the associated pain would be more intense compared to that of knee arthroscopic surgery [23]; therefore, all TKA patients should require a fairly similar dose of sevoflurane, despite preoperative ACB being performed. Second, ACB does not provide analgesia over the whole knee, as it targets the saphenous nerve and the nerve to the vastus medialis, which contributes to the innervation of the anteromedial knee joint [24]. On the other hand, our results also showed that intraoperative opioid consumption was significantly lower in Group B than in Groups A and C. Hence, it is reasonable to assume that the conserved doses of sevoflurane in Groups A and C were at the expense of higher intraoperative opioid consumption in these two groups. In fact, all of the patients in this study were under BIS-guided general anesthesia, and the BIS score was maintained in the range of 40–60 to ensure adequate anesthesia depth in these patients. The higher dose of intraoperative opioids consumed by patients in Groups A and C was intended to suppress the inherent surgical-induced stress, although hypertension was the only available index to reflect the stress. This result supports the effectiveness of preoperative ACB in lowering intraoperative surgical stress induced by TKA. This was also supported by the fact that fewer patients in Group B developed intraoperative hypertension compared with patients in Groups A and C. This is particularly important for elderly patients with impaired cardiovascular function because preoperative ACB can facilitate a stable hemodynamic state during surgery. Previous reports have shown that a prolonged fluctuation of the mean blood pressure that is more than 35% from baseline is significantly associated with the occurrence of a postoperative stroke [25,26]. 

Patients in Group A required more opioids at the PACU than patients in Groups B and C; however, this difference was not significant. This may suggest that acute postoperative pain in TKA is severe and that some patients, even with ACB, may require opioids for such acute postoperative pain, as ACB could not offer a completely pain-free knee and leave the posterior and lateral aspects of the knee unprotected. Another interesting finding regarding patient satisfaction for ACB upon discharge from PACU was that patients who received postoperative ACB (Group B) had higher satisfaction scores than patients in Groups A and C. This finding was not in accordance with the general concept of the preemptive nature of analgesic drugs for postoperative pain treatment [27,28]. Patients who received postoperative ACB showed a higher appreciation of the block, further supporting the effectiveness of ACB in lowering acute postoperative pain. It is reasonable to speculate that patients with preoperative ACB had a lower appreciation of ACB because they did not have an internal control of no ACB, as in Group C patients.

Encouragement of early mobilization is an important strategy to avoid postoperative knee stiffness and other complications after TKA [29]. Continuous passive motion (CPM) is an adjunct therapy for the initiation of early mobilization of the operated knee in patients that underwent TKA [30,31]. Adequate postoperative pain management encourages patients to participate in the CPM rehabilitation program [32] and facilitates a faster recovery to normal activities [33]. In addition, our study found that more patients in Group A quit their first CPM training because of intolerable pain after the first few cycles of passive motion training; these patients also had higher VAS scores than patients with ACB. Early mobilization is particularly important for elderly patients to avoid postoperative knee stiffness and a second surgery. Satisfaction after TKA for OA is usually high because of its cost-effectiveness [34] and high success for symptomatic and functional improvement [35]. Our study further supports the cost-effectiveness of ACB due to the reduction in the length of hospitalization. 

There are a few limitations to our study. First, our study may have suffered from potential bias inherent to retrospective studies. Second, stress hormones and cytokines, indicators of stress to trauma and surgery [36], were not measured in this study. Selected hormonal responses to surgery have been reported to reflect the degree of surgical stress [37]. It is reasonable to speculate that patients without ACB and patients with preoperative ACB should have a significant difference in surgery-induced hormonal values between these two groups of patients. Third, objective monitoring of intraoperative nociception was not available in this study, justifying the higher intraoperative consumption of opioids in Groups A and C patients, as the presumed nociception may be biased. Since two of the possible sources of intraoperative hypertension are surgical-induced nociception and reflectively enhanced sympathetic activity, objective nociception monitoring [38] is crucial to differentiate between these two sources. Fourth, long-term follow-up for patients with or without ACB who developed chronic pain was not available in this study. 

We concluded that, first, both preoperative and postoperative ACB reduced the postoperative VAS scores and opioid consumption at the PACU and the ward, as well as led to a shorter length of hospital stay. Second, preoperative ACB could be a better choice for patients undergoing TKA as it decreases intraoperative opioid consumption and facilitates a stable hemodynamic state during surgery. Third, further studies are necessary to verify our results, especially involving the measurements of stress hormones and objective nociception monitoring during surgery. 

## Figures and Tables

**Table 1:** Demographic characteristics of patients who underwent primary total knee arthroplasty under bispectral index-guided sevoflurane anesthesia, without adductor canal block (ACB) (Group A), with preoperative ACB (Group B), and with postoperative ACB (Group C).

Variables	Unit	N(%)/Median (IQR)	Group A (without ACB)	Group B (Preoperative ACB)	Group C (Postoperative ACB)	*p* Value
Sex	Female Male	478 (73.3%) 174 (26.7%)	200 (71.2%) 81 (28.8%)	165 (74.3%) 57 (25.7%)	113 (75.8%) 36 (24.2%)	0.533
Age	years	70.0 (65.0–75.0)	70.0 (64.0–74.5)	70.0 (65.0–75.0)	70.5 (64.5–76.0)	0.093
Weight	kg	67.0 (59.0–76.0)	67.0 (60.0–77.0)	67.0 (59.0–77.0)	67.0 (59.0–74.0)	0.319
ASA Physical Status	I II III	1 (0.2%) 401 (61.5%) 250 (38.3%)	0 (0.0%) 165 (58.7%) 116 (41.3%)	0 (0.0%) 133 (59.9%) 89 (40.1%)	1 (0.7%) 103 (69.1%) 45 (30.2%)	0.123
Anesthesia Time	hour	3.08 (2.83–3.50)	3.09 (2.88–3.58)	3.08 (2.82–3.45)	3.08 (2.80–3.50)	0.079
Hypertension	Yes	392 (60.1%)	174 (61.9%)	131 (59.0%)	87 (58.4%)	0.711
Diabetes	Yes	158 (24.2%)	76 (27.0%)	54 (24.3%)	28 (18.8%)	0.164
COPD	Yes	4 (0.6%)	2 (0.7%)	1 (0.5%)	1 (0.7%)	0.925
CAD	Yes	16 (2.5%)	10 (3.6%)	2 (0.9%)	4 (2.7%)	0.120
CHF	Yes	1 (0.2%)	0 (0.0%)	1 (0.5%)	0 (0.0%)	0.342
CVA	Yes	11 (1.7%)	6 (2.1%)	2 (0.9%)	3 (2.0%)	0.497
ESRD	Yes	12 (1.8%)	6 (2.1%)	5 (2.3%)	1 (0.7%)	0.404
Cancer	Yes	34 (5.2%)	18 (6.4%)	9 (4.1%)	7 (4.7%)	0.474

Numerical values are expressed as median (interquartile range) or number (%). COPD, chronic obstructive pulmonary disease; CAD, coronary artery disease; CHF, congestive heart failure; CVA, cerebral vascular accident; ESRD, end-stage renal disease.

**Table 2:** Intraoperative and postoperative presentations of patients who underwent knee arthroscopic surgery under bispectral index-guided sevoflurane anesthesia without adductor canal block (ACB) (Group A), with preoperative ACB (Group B), and with postoperative ACB (Group C).

Variables	Unit	N(%)/Median (IQR)	Group A (without ACB)	Group B (Preoperative ACB)	Group C (Postoperative ACB)	*p* Value
Intraoperative						
Sevoflurane	mL/kg/h	0.20 (0.17–0.25)	0.21 (0.17–0.26)	0.20 (0.17–0.25)	0.21 (0.17–0.26)	0.519
Opioid (MME)	mg/kg/h	0.074 (0.056–0.095)	0.078 (0.059–0.098)	0.065 (0.048–0.088)	0.078 (0.062–0.092)	<0.001
↑>30% SBP	Yes	399 (61.2%)	190 (67.6%)	117 (52.7%)	92 (61.7%)	0.003
PACU						
Opioid (MME)	mg/kg	0.0 (0.0–0.044)	0.0 (0.0–0.048)	0.0 (0.0–0.043)	0.0 (0.0–0.040)	0.089
Pain VAS	0–10	3.0 (2.0–3.0)	4.0 (4.0–4.0)	2.0 (2.0–2.0)	1.0 (1.0–1.0)	<0.001
Satisfaction	1–5	5.0 (4.0–5.0)	4.0 (4.0–4.0)	4.0 (4.0–5.0)	5.0 (4.0–5.0)	<0.001
In Ward						
Opioid (MME)	mg/kg	0.118 (0.107–0.129)	0.134 (0.113–0.156)	0.110 (0.101–0.139)	0.104 (0.087–0.121)	0.045
Parecoxib 40 mg	Yes	636 (97.5%)	271 (96.4%)	218 (98.2%)	147 (98.6%)	0.179
Pain VAS at Rest	0–10	2.0 (1.0–3.0)	3.0 (3.0–5.0)	2.0 (1.0–2.0)	1.0 (1.0–2.0)	<0.001
Pain VAS at CPM	0–10	2.0 (2.0–3.0)	3.0 (3.0–5.0)	2.0 (2.0–3.0)	2.0 (1.0–2.0)	<0.001
Intolerant CPM Pain on 1st Time		188 (28.2%)	98 (34.9%)	60 (27.0%)	30 (20.1%)	0.004
Satisfaction	1–5	5.0 (4.0–5.0)	5.0 (4.0–5.0)	5.0 (4.0–5.0)	5.0 (4.0–5.0)	0.95
Length of Stay	days	4.0 (3.5–5.0)	4.5 (3.5–5.0)	4.0 (3.5–5.0)	4.0 (3.5–5.0)	0.006
Dizziness	Yes	49 (7.5%)	22 (7.8%)	16 (7.2%)	11 (7.4%)	0.964
PON	Yes	30 (4.6%)	15 (5.3%)	9 (4.1%)	6 (4.0%)	0.737
POV	Yes	87 (13.3%)	44 (15.7%)	22 (9.9%)	21 (14.1%)	0.162

Numerical values are expressed as median (interquartile range) or number (%). MME, milligram morphine equivalent; CPM, continuous passive movement; PON, postoperative nausea; POV, postoperative vomiting.

## Data Availability

The data presented in this study are available from the corresponding authors upon reasonable request.
